# Evaluation of Quality Production Parameters and Mating Behavior of Novel Genetic Sexing Strains of the Mediterranean Fruit Fly *Ceratitis capitata* (Wiedemann) (Diptera: Tephritidae)

**DOI:** 10.1371/journal.pone.0157679

**Published:** 2016-06-23

**Authors:** Polychronis Rempoulakis, Gustavo Taret, Ihsan ul Haq, Viwat Wornayporn, Sohel Ahmad, Ulysses Sto Tomas, Thilakasiri Dammalage, Keke Gembinsky, Gerald Franz, Carlos Cáceres, Marc J. B. Vreysen

**Affiliations:** 1 Insect Pest Control Laboratory, Joint FAO/IAEA Division of Nuclear Techniques in Food and Agriculture, International Atomic Energy Agency, Vienna, Austria; 2 Department of Biological Sciences, Macquarie University, North Ryde, NSW 2109, Australia; 3 National Agricultural Research Centre, Islamabad, Pakistan; CNRS, FRANCE

## Abstract

The Mediterranean fruit fly *Ceratitis capitata* (Wiedemann) (Diptera: Tephritidae) is one of the most important pest of fruits and vegetables in tropical and subtropical countries. The sterile insect technique (SIT) as a component of area-wide integrated pest management (AW-IPM) approaches is being used for the successful management of this pest. VIENNA 8 is a genetic sexing strain (GSS) that has a white pupae (*wp*) and temperature sensitive lethal (*tsl*) mutation, the latter killing all female embryos when eggs are exposed to high temperatures (34°C). The use of this GSS permits production and the release of only males which has increased the cost effectiveness of the SIT several fold for this pest. An efficient method of identification of recaptured sterile males can further increase the cost effectiveness of the SIT for this pest. Therefore, VIENNA 8-Sergeant2 (Sr^2^) strain and the transgenic strain VIENNA 8–1260 having visible markers were constructed. All three strains were evaluated for egg production, egg hatch, and egg sterility parameters under semi mass-rearing conditions and mating competitiveness in field cages. VIENNA 8–1260 females produced significantly fewer eggs as compared with the two other strains, which produced similar numbers of eggs. However, egg hatch of all strains was similar. Egg hatch of eggs produced by untreated females that had mated with adult males that had been irradiated with 100 Gy as pupae 2 days before emergence, was different for the three strains, i.e., egg hatch of 0.63%, 0.77%, 0.89% for VIENNA 8, VIENNA 8–1260, and VIENNA 8-Sr^2^, respectively. Differences in male mating competitiveness of the three strains against wild-type males were gradually reduced with successive generations under semi mass-rearing conditions. However, VIENNA 8 males adapted faster to laboratory conditions as compared with VIENNA 8-Sr^2^ and VIENNA 8–1260 males with respect to mating competitiveness. VIENNA 8 males of the F_10_ generation were equally competitive with wild-type males, whereas the mating competitiveness of VIENNA 8-Sr^2^ and VIENNA 8–1260 males was similar but lower as compared with wild-type males. Males from all three strains copulated earlier than wild-type males. Results are discussed in relation with the potential benefits of incorporating novel strains for more effective SIT application.

## Introduction

The Mediterranean fruit fly *Ceratitis capitata* (Wiedemann) (Diptera: Tephritidae) is the most destructive fruit fly pest in the world, causing extensive damage to fruits and vegetables [1,2]. It is extremely polyphagous, and attacks more than 350 species of fruits and vegetables belonging to more than 67 plant families [3,4]. Control methods employed against it include aerial and ground sprays, the use of variety of food attractants and other semiochemicals for lure and kill of both sexes [5–7]. The sterile insect technique (SIT) is an eco-friendly method for the management of selected insect pests. It relies on the mass-rearing of insects of the target population, sterilizing them by ionizing radiations and release of the sterile males in the target area where they will mate with virgin wild females and transfer their sterile sperm which results in unviable eggs [8]. Successive releases of sterile insects will gradually reduce the density of the target population to a very low, economically acceptable level and in some cases, eradication might be achievable [8]. The SIT has been proven to be very effective for the suppression, containment, prevention or eradication of populations of the Mediterranean fruit fly [9]. Two main factors have made the SIT for the Mediterranean fruit fly more cost-effective, i.e. the first was the technological developments that enabled the production of the flies on a large scale and the second was the development of genetic sexing strains (GSS) that enabled male-only releases [9]. Testimony to this is the fact that the overwhelming majority of operational programmes against this pest in the world that have an SIT component are rearing the GSS of this species [10]. The use of GSS revolutionized the SIT application in many ways: (1) male-only releases reduced the cost of rearing and releasing sterile males, (2) male-only releases eliminated the assortative mating between sterile male and females, making the SIT component much more effective, and (3) dispersal of the sterile males was increased which enabled them to transfer their sterile sperms to more wild females. Releasing sterile males only induced a higher level of sterility in the target population as compared with bi-sex releases [11,12]. Furthermore, damage to the fruit that occurs due to oviposition by sterile females and secondary infestations caused by microbes can be avoided [13].

The development of a GSS for the Mediterranean fruit fly has undergone some distinct phases, starting with the so called ‘first generation GSS’ which was based on a pupal color mutation. This system permitted the separation of males (wild-type brown pupae) from females (mutant white pupae) by optical means [14–16]. However, this system was not 100% accurate and the releases of few sterile females could cause damage to fruits in commercial fields [17]. In addition the strain did still require the rearing of all female larvae which incurred a significant cost. Therefore, more advanced genetic sexing systems that enabled the separation of the sexes at the embryonic stage (thus avoiding the high cost of rearing female larvae) were desirable. During the last forty years, the Joint FAO/IAEA Sub-programme of Nuclear Techniques in Food and Agriculture coordinated research by groups from different parts of the world for the development of ‘second generation GSS’ that were mainly based on a *temperature sensitive lethal* (*tsl*) mutation. The *tsl* mutation allowed for early elimination of the female flies (exposure of eggs to 34°C for 24 h kills all female embryos) and larval rearing of male only lines for release in the field, whereas thermally untreated embryos from both sexes of such strains could be reared for colony propagation. The strain based on the *tsl* mutation (VIENNA 8) has proven to be very stable and has been adopted by all Mediterranean fruit fly mass-rearing facilities in the world [18].

In addition to cost-effective mass-rearing of sterile males, each programme that incorporates the SIT component requires an efficient monitoring system to assess sterile:wild male over-flooding ratios, rate of induced sterility in the wild population, distribution of the released sterile males in space, apparent densities of the wild target population and sterile male survival etc. [19]. Monitoring however, is expensive and can cost up to one third of the entire programme budget [11], and therefore, marking techniques for the sterile males are needed which are both accurate and reliable. All these monitoring parameters are important indicators for the success of the SIT component of the programme, and provide decisive feed-back information for programme managers to take corrective measures, such as the increase of released individuals per unit area and/or changes in release schedule and methods [19].

The most commonly used marking system for Mediterranean fruit flies relies on the dying of pupae with fluorescent powder which gets sequestered in the frontal suture or ptilinal suture of adults during their emergence from the puparium [20,21]. This technique has been proven very reliable and cost effective [22]. However, in the event that sterile flies are detected without the presence of fluorescent powder, the false diagnosis of wild males will result in a prolongation of the control efforts (at least for eradication strategies) which will increase programme’s cost. Therefore, research has been conducted to explore new innovative options to replace the fluorescent dye marking system. In that respect, two new Mediterranean fruit fly strains were constructed that were both based on the VIENNA 8 strain’s genetic background but also carried a visible marker. The VIENNA 8-Sr^2^ strain was constructed using Mendelian genetics that carried the dominant Sergeant 2 (Sr^2^) mutation on the autosome-5 while the females of this strain are wild-type with respect to this marker [23,24]. The released males of this strain can be easily distinguished from the wild flies in the target population with the naked eye by the presence of three abdominal stripes as compared with two stripes of the wild strain. The second novel strain (VIENNA 8–1260) is a transgenic strain that expresses red fluorescence of the body and green fluorescence of the testes and the sperm [25]. The marked sperm not only facilitates identification of trapped males but can also be used to estimate mating success of the released sterile males and hence, the rate of induced sterility in the wild female population in the target area.

The objectives of the present study were to evaluate the productivity of these three strains (fecundity and fertility) under semi mass-rearing conditions, the mating competitiveness of the mass-reared males against wild-type (bisexual strain) males and their ability to induce sterility to wild-type females under semi-natural conditions.

## Materials and Methods

### Strains

#### VIENNA 8

VIENNA 8 is a GSS that carries a *tsl* mutation and that was developed at the FAO/IAEA Insect Pest Control Laboratory (IPCL), Seibersdorf, Austria. This strain has two mutations with two markers *wp* and *tsl* on Y-autosome 5 [18]. For the series of experiments, the strain was freshly constructed by crossing the males from the parental stock of VIENNA 8 GSS with females from the parental stock that carry in homozygous conditions the *tsl* mutation and *wp* mutation. The F_1_ males were again crossed with females from the *tsl* and *wp* parental stock to have a pure genetic background of VIENNA 8 *tsl wp*.

#### VIENNA 8-Sr^2^

Vienna 8-Sr^**2**^ is a GSS that was developed at the FAO/IAEA IPCL and that carries a *tsl*, a *wp*, and one additional mutation, i.e. the marker Sr^2^ that is situated very close to the *tsl* gene [24]. The strain used in the experiments was also newly constructed using the same procedure as with the VIENNA 8 GSS *tsl wp*.

#### VIENNA 8–1260

VIENNA 8–1260 is a transgenic strain developed by collaborators from the University of Göttingen, Germany and the University of Pavia, Italy. This strain was developed by backcrossing females from transgenic testes-marked strain #1260 with male VIENNA 8. The 1260 transgenic strain expresses Ccb2t promoter-driver tGFP in the testes and DsRed in the body [25]. The strain used in the experiments was also freshly constructed by using the same procedure as with the VIENNA 8 GSS *tsl wp*.

In conclusion, all three strains were newly constructed and carried the same genetic background as VIENNA 8 GSS *tsl wp* with additional markers present in VIENNA 8-Sr^2^ and the transgenic VIENNA 8–1260 strain.

#### Wild-type strain

The wild-type strain that was used in the experiments, originated from pupae collected in Argentina and had been reared at the IPCL for 25 generations at the time of the initiation of the experiments. This strain does not carry any morphological marker, or other mutation, and it was used as reference for the mating behavioral assessment of the other three strains. We selected this strain that was well domesticated at the beginning of the experiments, to enable comparisons of the behavior of the three GSS with the wild-type strain over a significant period of time (i.e.10 generations or one year) without the risk that the effects of the domestication of the wild type strain would interfere with our results.

### Rearing protocol

The three Mediterranean fruit fly GSS were reared under a semi mass-rearing environment at the IPCL. Around 300,000 pupae at a 1:1 male:female ratio were placed in standard mass-rearing cages that had an aluminum frame (201 (length) × 100 (height) × 20.5 cm (width)) with both sides covered with muslin cloth net for females oviposition. These cages were hung from the roof side-by-side, 200 cm from the wall and 100 cm apart (each cage was suspended half-way between lights) in a room at 24±1°C and 60±5% RH with photoperiod 12:12 D/L. Adults were provided with a diet containing hydrolysate yeast as a source of protein and sugar in a ratio of 1:3 by weight respectively and water *ad libitum*. Females laid eggs by inserting their ovipositor through the muslin cloth net. Eggs were collected in galvanized iron troughs containing water that were placed at the bottom of these mass-rearing cages. Eggs were sieved out daily from the water and incubated in aerated water for 48 h at 24°C, but for the mating competitiveness trials that required only male flies, the eggs were first aerated for 24 h at 24°C and thereafter “bubbled” in water at 34°C for another 24 h to eliminate the female embryos [26,27]. After incubation the eggs were transferred to a standard larval diet [28] that contained wheat bran as the bulking agent. A volume of 3.5 mL of eggs (homogeneously mixed in water) was transferred to 3 kg larval diet in standard plastic larval Hawaiian rearing trays (77 x 40 x 3.8- cm (length—width—height) and a height of 7 cm at the corners). The difference in height of the sides and corners of the trays allows stacking them on top of each other leaving a gap of 3.2 cm between the surface of the diet in one tray and the bottom of the next tray, providing essential ventilation for the developing larvae and adequate space for the mature larvae to “pop out” of the diet for pupation [29]. Larvae that had left the diet were collected in saw dust in metal trays placed at the bottom of larval trays. The same rearing protocol was used for all three GSS that were reared in parallel and in the same insectary. The three strains were maintained under semi mass-rearing conditions for 10 generations, each one lasting approximately one month.

The wild-type flies were reared in less crowded (relaxed) conditions in 30 x 30 x 45 cm plexiglass cages that had openings for manipulations that were covered with muslin cloth. The adult feeding protocol was the same as for the GSS and eggs were collected in small wax domes that mimicked fruits. The larval diet was also the same as the one used to feed the GSS larvae, but the larval plastic trays were smaller (30 x 40 cm).

### Egg production

During the 10 generations of semi mass-rearing, the volume of the daily egg collections was recorded. Eggs produced by each generation were collected for 2 weeks and their volume and hatch rate were assessed. Egg productivity data was recorded for eight generations. Egg productivity per female was roughly estimated by dividing the number of produced eggs by the number of white pupae (only females emerged from white pupae) placed in the mass-rearing cages. A sample of ~1000 eggs from each generation was taken for assessing egg hatch.

### Radiation sterility assessment

Pupae were exposed to 100 Gy radiation 2 days before adult emergence in a Co^60^ Gamma Cell 220 (Nordion, Canada) irradiator. Dosimetry of the source was performed following the Gafchromic dosimetry system (Gafchromic HD-810 film, International Specialty Products, Wayne, NJ). Three 10 x 10 mm film dosimeter were placed in a small paper envelope, which was placed among the pupae before irradiation. The optical density measurements were performed on a Radiochromic reader (FWT-92D, Far West Technology, Inc., Goleta, CA) 24 h after irradiation, and the dose was calculated according to IAEA (2004). Males emerging from irradiated pupae and non-irradiated males were allowed to mate with untreated and treated females and egg sterility was observed by measuring egg hatch. For comparison of egg sterility, all 4 possible combinations between sterile and fertile males and females were tested as follows: sterile males were allowed to mate with sterile and fertile females separately and fertile males with sterile and fertile females separately. For this purpose 10 couples from each combination were placed in a small screened plexiglass cage and provided with protein diet and water *ad libitum*. Females oviposited eggs through the screen cloth that were collected in Petri dishes for 10 days during the 3^rd^, 5^th^, 6^th^, 7^th^, and 10^th^ generation. Daily collected eggs were counted and placed on a wet sponge and the number of unhatched eggs was counted on the fifth day following egg collection. Egg sterility was calculated as percentage of unhatched eggs.

### Field cages

The field cages used for the mating competitiveness trials were standard screened, circular field tents (2.2 m high × 2 m diameter) [30,31], containing a small potted citrus tree of ~2 m height. Nine field cages were placed inside a large insect greenhouse (24 × 10 × 4 m) and a temperature of 23 ± 2°C and 60% ± 5% RH was maintained throughout the experiments. Field cage tests took place under a semi-natural illumination and photoperiod provided by the translucent roof of the insect greenhouse and ten replications were evaluated.

### Mating competitiveness tests in field cages

Mating competitiveness tests were performed in field cages adopting the standard FAO/IAEA/USDA protocols [31]. From each strain, a sample of ~300 pupae was taken from the F_3_, F_6_, and F_10_ generations maintained under semi mass-rearing conditions and transferred to cylindrical plexiglass screened cages (30 cm height and 64 cm diameter) and kept in the insectary where the wild-type flies were maintained. Wild type flies were sexed 1–2 days after emergence and males and females maintained in separate cages. All flies of all strains that were used for the mating competitiveness studies were kept at insectary conditions of 23°C and 65 ± 5% RH and were provided with a protein diet and water *ad libitum*. Males from each strain were marked with a different water color using a camel brush and the mark was applied on the dorsal side of the thorax of males confined by a screen net. Male marking was done 1–2 days before the mating trials. Fifty males of each strain competed for fifty wild females at a male: female ratio of 2:1. All males were released in field cages early in the morning after dawn and allowed to establish territory, and 20 minutes later the females were released, and allowed to complete copulation under observation (every 5 min). The recorded parameters were number of matings, male type, mating latency, mating duration, location of couples (tree canopy or field cage), elevation (top, middle, or lower part of the tree canopy) and position of couples (upper side of leaf or lower side of leaves).

For every replicate of each experiment, in conjunction with the competitiveness field cage tests, we tested the general mating propensity of each one of the four strains i.e. the 3 GSS and the wild-type one by containing 50 males and 50 females from the same strain in a no choice cage.

### Data Analysis

Mating competitiveness of the experimental males was assessed using the Relative Sterility Index (RSI), and was estimated by the formula [31]:

$$RSI=\frac{SW}{SW+WW}$$

with SW denoting matings of sterile males with wild females; WW denoting matings of wild males with wild females. Values of RSI can vary from 0 to 1, where 0 indicates that all of the wild females that mated in the cage mated with wild males, 1 indicates that they all mated with sterile males, and 0.5 indicates that half mated with sterile males and half mated with wild males and that sterile males are equally competitive with wild males [31]. Chi-square analysis was performed to analyze the equal propensity of mating between sterile and wild males.

Mating latency was calculated as the time elapsed from the release of females till initiation of a given mating. Mating duration was calculated as the time when mating pairs separated (i.e. no longer physical contact) minus the time at which they started to mate.

Data expressing percentage (egg hatch data) were arcsine transformed prior to analysis. Analysis of variance was used to analyze data of egg production, mating duration, and mating latency. Complementary pair-wise comparison (Tukey-Kramer HSD test) was performed for treatments that were significantly different from each other. A Kruskal-Wallis nonparametric analysis was performed for data of % egg hatch. The statistical program SAS Jump for windows was employed for the analysis of the data.

## Results

### Egg productivity under semi mass-rearing conditions

**Fig 1:**
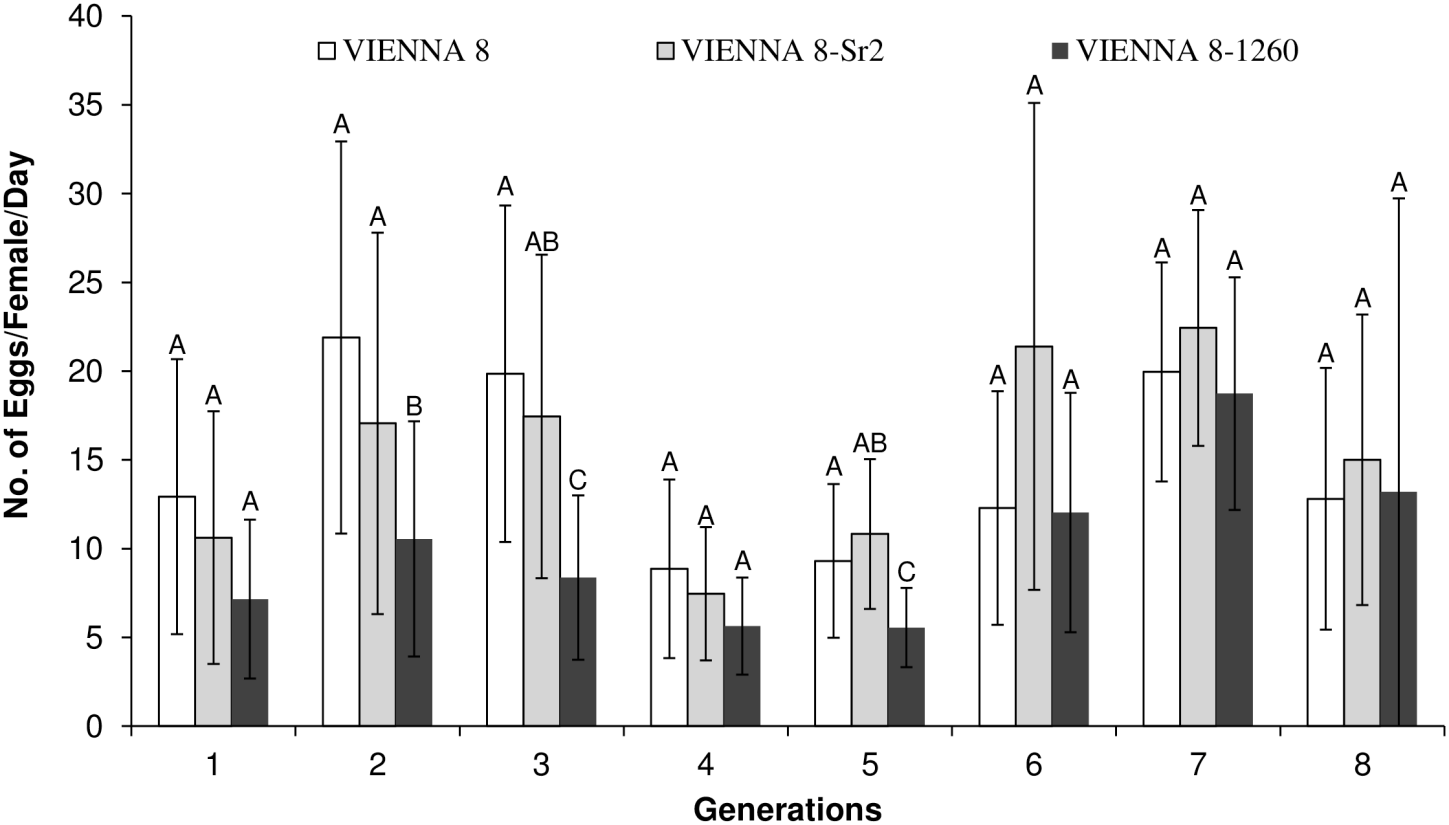
Number of eggs (± SD) produced per female per day by the Mediterranean fruit fly genetic sexing strains VIENNA 8, VIENNA 8-Sr^2^ and VIENNA 8–1260 under semi mass-rearing conditions. Means followed by the same letter do no differ significantly (Tukey-Kramer HSD, α = 0.05).

**Fig 2:**
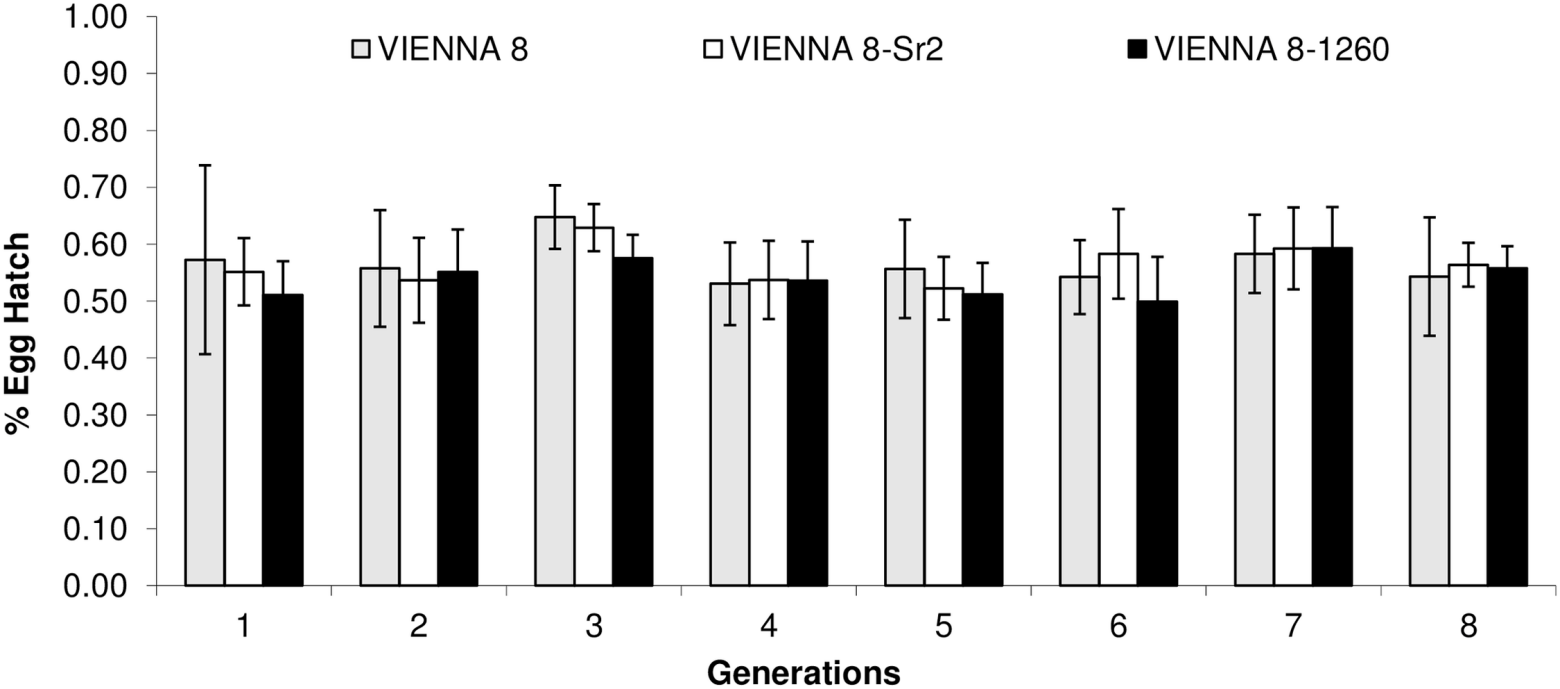
Mediterranean fruit fly genetic sexing strains VIENNA 8, VIENNA 8-Sr^2^ and VIENNA 8–1260 egg hatch (% ± SD) data during the course of eight generations under semi mass-rearing conditions.

Comparison of egg production per female per day of the 3 strains showed significant differences for the 2^nd^ (F_2,38_ = 4.49, *P* = 0.01), 3^rd^ (F_2,29_ = 5.66, *P* = 0.008) and 5^th^ (F_2,41_ = 7.46, *P* = 0.002) generation, but no significant differences were found in egg production for the 1^st^, 4^th^, 6^th^, 7^th^ and 8^th^ generation. However, total egg production during the eight generations of the experiment showed significant differences among the 3 strains (F_2,265_ = 7.74, *P* = 0.0054), with VIENNA-8 and VIENNA-8.Sr^2^ producing equal numbers of eggs (14.6 and 14.9 eggs/female/day, respectively), but VIENNA 8–1260 produced significantly fewer eggs (9.8 eggs/female/day) (Fig 1). With respect to the hatch rate of eggs collected from the mass-rearing cages, the three strains did not differ (F_2,207_ = 3.05, *P* = 0.079) from each other and also they exhibited similar egg hatch rates during the 8 generations tested (Fig 2).

### Radiation sterility

Among the 4 different irradiation combinations (Fertile male X Fertile female, Sterile Male X Fertile female, Fertile male X sterile female, Sterile male X Sterile female), eggs were only produced in those matings that involved fertile (non-irradiated) females. Eggs collected during the course of the 5 generations tested (3^rd^, 5^th^, 6^th^, 7^th^, 10^th^) revealed that the 3 strains had significantly different egg hatch rates in those combinations with non-irradiated males (F_2,887_ = 27.5, *P* < 0.001), i.e. the eggs produced by the VIENNA 8–1260 strain had a significantly lower hatch rate than the other 2 strains (egg hatch of 66.7%, 65.5%, 58.8% for VIENNA 8, VIENNA 8-Sr^2^ and VIENNA 8–1260, respectively). When irradiated males were used in the crosses with non-irradiated females, again significant differences were observed between the strains, with the lowest egg hatch observed for eggs produced by the VIENNA 8 strain, followed by VIENNA 8–1260 and VIENNA8-Sr^2^ (respective values of 0.63%, 0.77%, 0.89%). Detailed results from the different generations and mating combinations of the 3 strains are presented in Table 1.

**Table 1:** Egg hatch (%) of three strains of Mediterranean fruit fly, presenting the effect of irradiation on males and females at different generations under mass-rearing conditions (10 days of egg collection for each generation were tested for successful hatching). For the sterile sex of each one of the following combinations, the sterilizing dose was 100 Gy delivered from Co^60^ source. Average and standard deviation of egg hatch percentage are presented, total number of eggs examined are presented in parenthesis.

Combinations	Strains	Generations				
		3 ^rd^	5^rth^	6^th^	7^th^	10^th^
**Fertile male X Fertile female**	**VIENNA 8**	63.5±8.0 (**23490**)	57.4±10.6 (**26815**)	62.6±9.1 (**21465**)	60.9±7.9 (**22466**)	64.2±11.2 (**10622**)
	**VIENNA 8-Sr**^**2**^	61.8±7.7 (**22617**)	56.7±11.2 (**26481**)	61.7±11.1 (**15026**)	59.3±9.5 (**18766**)	64.2±9.7 (**10513**)
	**VIENNA 8–1260**	56.7±9.5 (**25233**)	53.8±11.8 (**26271**)	44.4±22.0 (**16301**)	60.0±8.9 (**17564**)	62.2±8.9 (**12607**)
**Sterile male X Fertile female**	**VIENNA 8**	0.6±0.5 (**21073**)	0.3±0.24 (**29720**)	0.6±0.6 (**20011**)	0.7±0.7 (**24293**)	1.1±0.9 (**12310**)
	**VIENNA 8-Sr**^**2**^	0.7±0.5 (**15932**)	0.4±0.3 (**20694**)	0.9±0.9 (**14246**)	0.9±0.9 (**16004**)	1.7±0.1 (**9309**)
	**VIENNA 8–1260**	0.5±0.3 (**27081**)	0.4±0.2 (**28518**)	0.7±0.8 (**16509**)	0.7±0.6 (**17031**)	1.7±1.5 (**13038**)
**Fertile male X Sterile female**	**VIENNA 8**	N/A (**0**)	N/A (**0**)	N/A (**0**)	N/A (**0**)	N/A (**0**)
	**VIENNA 8-Sr**^**2**^	N/A (**0**)	N/A (**0**)	N/A (**0**)	N/A (**0**)	N/A (**0**)
	**VIENNA 8–1260**	N/A (**0**)	N/A (**0**)	N/A (**0**)	N/A (**0**)	N/A (**0**)
**Sterile male X Sterile female**	**VIENNA 8**	N/A (**0**)	N/A (**0**)	N/A (**0**)	N/A (**0**)	N/A (**0**)
	**VIENNA 8-Sr**^**2**^	N/A (**0**)	N/A (**0**)	N/A (**0**)	N/A (**0**)	N/A (**0**)
	**VIENNA 8–1260**	N/A (**0**)	N/A (**0**)	N/A (**0**)	N/A (**0**)	N/A (**0**)

### Male mating competitiveness under field cage conditions

The mating competitiveness of the three strains was evaluated under field cage conditions by testing males from the F_3_, F_6_ and F_10_ generations which were reared under semi mass-rearing conditions. F_3_ generation males of the VIENNA 8 and VIENNA 8–1260 strains were found less competitive as compared with the wild-type males but still showed somehow satisfactory mating performance (RSI = 0.29, RSI = 0.32 respectively). However, F_3_ generation males from the VIENNA 8-Sr^2^ strain were the least competitive with a RSI value of 0.09. Disparity in mating competiveness of the males of all three strains against wild-type males reduced during the process of domestication but in the sixth generation, the males of all three GSS remained less competitive than the wild-type males (RSI of 0.36, 0.34, 0.41 for males of VIENNA 8, VIENNA 8-Sr^2^, and VIENNA 8–1260, respectively; Chi-squared *P* < 0.05 for all three strains). However, in the tenth generation, mating competiveness of the males of the VIENNA 8 strain was similar to that of the wild-type males (RSI = 0.51; Chi-squared *P* > 0.05), but the mating competitiveness of the VIENNA 8-Sr^2^ and VIENNA 8–1260 males of the F_10_ generation remained significantly lower than that of the wild-type males (RSI of 0.43 and 0.42, respectively; Chi-squared *P* < 0.05 for both strains) (Table 2).

**Table 2:** RSI indices from mating competitiveness experiments with three laboratory strains of the Mediterranean fruit fly tested against a wild-type Argentina population in field cages. Couples observed for each type of mating, RSI values± SD with chi-square results (*p* > 0.05) showed equal mating propensity. Number of couples from each strain followed by same letter are not significantly different from each other (Tukey-Kramer HSD, α = 0.05) and numbers of cages that have been taken into account (>50% of total expected) in parenthesis.

Generations					
3 ^rd^		6^th^		10^rth^	
**VIENNA 8 male X Wild-type male**					
**VIENNA 8 male**	**Wild-type male**	**VIENNA 8 male**	**Wild-type male**	**VIENNA 8 male**	**Wild-type male**
54 ^b^ RSI 0.29 ± 0.16	102^a^ *p* = 0.000	92 ^b^ RSI 0.36 ± 0.06	161^a^ *p* = 0.000	203^a^ RSI 0.51 ± 0.10	187^a^ *p* = 0.418
**VIENNA 8-Sr**^**2**^ **male X Wild-type male**					
**VIENNA 8-Sr**^**2**^ **male**	**Wild-type male**	**VIENNA 8-Sr**^**2**^ **male**	**Wild-type male**	**VIENNA 8-Sr**^**2**^ **male**	**Wild-type male**
17 ^b^ RSI 0.09 ± 0.05	107^a^ *p* = 0.000	47 ^b^ RSI 0.34 ± 0.10	95^a^ *p* = 0.000	154 ^b^ RSI 0.43 ± 0.06	209^a^ *p* = 0.004
**VIENNA 8–1260 X Wild-type male**					
**VIENNA 8–1260 male**	**Wild-type male**	**VIENNA 8–1260 male**	**Wild-type male**	**VIENNA 8–1260 male**	**Wild-type male**
69 ^b^ RSI 0.32 ± 0.15	113^a^ *p* = 0.001	110 ^b^ RSI 0.41 ± 0.13	164^a^ *p* = 0.001	146 ^b^ RSI 0.42 ± 0.12	193^a^ *p* = 0.011

The mating latency (measured in minutes following release in the field cages) was shorter for all 3 experimental strains as compared with their wild-type counterparts (Table 3). The duration of mating was significantly shorter for males of the VIENNA 8-Sr^2^ strain as compared with males of the VIENNA 8, VIENNA 8–1260 and the wild-type strain (Table 3). In control cages that contained males and females of the same strain, VIENNA 8-Sr^2^ males showed again shorter mating duration (Table 3).

**Table 3:** Mating latency (min) and mating duration (min) among three laboratory strains of Mediterranean fruit fly and a wild-type Argentina strain. Average mating latency or duration ± standard deviation is presented, couples observed are presented in parenthesis. First column for each parameter presents results collected from competitiveness study cages, second column presents results collected from control cages. Within the columns, numbers followed by the same letter do no differ significantly (Tukey-Kramer HSD, α = 0.05).

Mating latency (min)			Mating duration (min)		
Male origin	Female origin		Male origin	Female origin	
	Wild-type	Lab		Wild-type	Lab
Wild-type	^a^91±66 (1616)	N/A	Wild-type	^a^168±54 (2159)	N/A
VIENNA 8	^c^70±50 (372)	^**b**^90±6 (497)	VIENNA 8	^a^167±57 (372)	^a^153±52 (497)
VIENNA 8-Sr^2^	^bc^71±51 (256)	^**b**^87±59 (417)	VIENNA 8-Sr^2^	^b^151±50 (256)	^b^134±41 (417)
VIENNA 8–1260	^ab^83±62 (376)	^**a**^99±65 (420)	VIENNA 8–1260	^a^164±57 (373)	^a^151±51 (420)

Fifty four percent of the couples that contained wild-type males were found mating on the host plants, whereas this percentage was significantly lower for the couples that contained males of the VIENNA 8, VIENNA 8-Sr^2^ and the VIENNA 8–1260 strain (43.7%, 38.6% and 30.9%, respectively). From those couples isolated on the host tree, most were collected from underside of leaves (91.1%, 92.4%, 94.9% and 88.7% for wild-type males, VIENNA 8, VIENNA 8-Sr^2^ and Vienna 8–1260 males respectively). Finally, most mating couples were collected from the top part of the field cages (~85%), with no differences between the different strains.

## Discussion

The VIENNA 8, Vienna 8-Sr^2^, and Vienna 8–1260 strains were reared under semi mass-rearing conditions for 10 generations, and the results showed that the Vienna 8 and the Vienna 8-Sr^2^ produced similar but significantly higher number of eggs per female than the Vienna-8-1260. However, there were no differences in the viability of the fertilized eggs produced by the 3 strains. Mating of untreated females with irradiated sterile males showed significant differences in induced sterility with the lowest egg hatch observed for females mated with Vienna 8 sterile males, followed by that of females mated with Vienna 8-Sr^2^ and Vienna 8–1260. Competitiveness of the early (generation F_3_) males from the three tested GSS was lower as compared with that of the wild-type males, with Vienna8-Sr^2^ male being the least competitive, whereas VIENNA 8 and Vienna 8–1260 males were equally competitive. In subsequent generations, the disparity in mating competitiveness of all GSS males in comparison with wild-type males gradually decreased and F_10_ males of the Vienna 8 strain were equally competitive with wild-type males. Competitiveness of Vienna 8- Sr^2^ and Vienna 8–1260 F_10_ males was similar but remained lower as compared with that of the wild-type males. In addition, GSS males showed lower mating latency as compared with wild-type males and most males tended to copulate in the upper parts of the field cage and under the leaves of the citrus trees.

Currently all area-wide integrated pest management (AW-IPM) programmes [9] against the Mediterranean fruit fly that are incorporating the SIT component are using genetic sexing strains [10]. Releasing only sterile males has drastically increased the cost-effectiveness of such action programmes and has increased the biological efficiency of the SIT component in many ways [11]. However, the GSS of the Mediterranean fruit fly that were constructed using classical Mendelian genetics had certain disadvantages. First, the productivity of the mass-reared colony was reduced to 50%. Secondly, sex-linked translocations are not stable and the GSS tended to break down during successive generations under mass-rearing [32]. To maintain the stability of genetic sexing strains under mass-rearing environment a filter rearing system (FRS) was developed [33]. The FRS is to maintain a small standby colony of GSS under less crowded (relaxed) conditions, with no recombinants, which can regularly refresh the mainstream of production with new material and the key factor is that no flies from the mainstream colony are to be returned to the filter colony. Additionally, Franz [18] suggested that stability in translocation based genetic sexing systems can be improved by incorporating inversions that covers the critical region between the translocation break point and the selectable markers. Thus, the VIENNA 8 strain was constructed by inserting inversion D53 along with translocation T(Y;5) 101 and as a result, this translocation increased fertility with 10–30% making this strain more suitable and stable for mass-rearing purposes [18,34]. The VIENNA 8-Sr^2^ strain was developed assuming that the Sr^2^ marker would have no effect on fertility of the strain. Our results support this assumption that this mutation had no fertility reducing effect on the females and VIENNA 8 and VIENNA 8-S^2^ had similar egg production and egg viability.

Here we provide the first report of an analysis of the fecundity of the transgenic VIENNA 8–1260 strain under semi mass-rearing conditions, and our data showed that fecundity of the transgenic VIENNA 8–1260 was lower as compared with that of VIENNA 8 and VIENNA 8-Sr^2^. Scolari et al [25] studied sperm storage, sperm count, sperm viability over time and egg hatch as indicators of the fertility of the VIENNA 8–1260 under laboratory rearing conditions and showed that the transgenic line transferred less sperm and had a lower egg hatch when compared with wild-type flies. However, sperm use pattern over time was similar in VIENNA 8–1260 as compared with that of wild-type flies. Our results corroborate these data and showed lower egg production and lower egg hatch of VIENNA 8–1260 females as compared with that of VIENNA 8 females, which raises concerns that mass-rearing of the transgenic line might increase production costs due to lower egg production of the females.

For the success of SIT the mass-reared sterile males should be able to effectively transfer sterile sperm to wild females [35]. Franz [36] reported that males of the *tsl* GSS are partially sterile due to a simple reciprocal translocation. Irradiation of male pupae 24 h before emergence with 80–90 Gy resulted in > 99% sterility (compared to 100 Gy required to obtain the same level of sterility in wild-type males) which is a great advantage as the lower radiation dose will induce less somatic damage which is expected to increase the competitiveness of the sterile males [35]. Our data showed that VIENNA 8 males were the most successful in inducing sterility into the wild-type females which seems to justify using the VIENNA 8 as the preferred strain for mass-rearing. Replacing the VIENNA 8 with another strain in an operational programme is an important decision and should only be taken by managers if the replacement strain has similar performance or major other benefits. The fact that the VIENNA 8–1260 males were less successful in inducing sterility into wild females that in addition to regulatory restrictions for transgenic strains releases in the field makes this strain a less than ideal candidate to replace the VIENNA 8 in mass-rearing facilities.

Another critical parameter for the success of SIT is the mating competiveness of mass-reared sterile males against wild males which is adversely affected due to the mass-rearing environment. Reduced mating competitiveness of mass-reared males that is mainly due to high crowding conditions and lower sexual selection pressure in a mass-rearing environment has been shown for the Mediterranean fruit fly and other fruit fly species [37,38]. Likewise, in this study, the 3^rd^ generation males of all three GSS strains were less competitive than their wild-type counterparts and this disparity in male mating competiveness was reduced gradually in successive generations under semi mass-rearing conditions. However, caution is needed in interpreting the results that mating competitiveness of males increases gradually with each generation. It seems obvious that the increase in mating competitiveness with time of the 3 GSS can be linked to the domestication process, but the VIENNA 8 males adapted faster to the rearing conditions than the other GSS tested. VIENNA 8 males reached a similar level of mating competiveness as wild-type males in the 10^th^ generation, whereas the VIENNA 8-Sr^2^ and VIENNA 8–1260 males remained significantly less competitive as compared with their wild-type counterparts. However, the issues that remain unanswered are: 1.) is the lower male mating competiveness of the VIENNA 8-Sr^2^ and VIENNA 8–1260 strains due to the deleterious effects of the markers, or 2.) due to the limited genetic diversity during the construction process of these strains, and more research is required to answer these questions. Furthermore, it is important to point out that our studies do not demonstrate which strain has potentially the best mating performance in the field, as this can only be demonstrated either in the field, or under semi-field conditions using wild insects. Mass-rearing seems affecting other parameter of mating behavior, i.e. mating latency also. In this study males from all three strains showed shorter mating latency as compared to wild-type males. This pattern was in accordance with the previous studies that showed that mass-reared males did initiate sexual activities earlier than wild counterparts. Calkins [39] argued that due to the high density of flies in the mass-rearing cages, males that initiate sexual activities earlier have a distinct advantage in securing matings as compared to those that mate later.

In addition to cost-effective mass-rearing of a target insect, the success of the SIT component of AW-IPM programmes can only be guaranteed if adequate sterile to wild male ratios are obtained in the monitoring traps [19]. Due to the polyandrous behaviour of the Mediterranean fruit fly [40] not only the number of male and female flies trapped is an important parameter but also mating status of wild females, i.e. having mated with a wild or a sterile male or both provides accurate info on the rate of induced sterility in the wild population and provides good feedback on the progress of the operational programme [19]. Giving less attention to monitoring can jeopardise the whole investment incurred in action programmes [19]. In view of the importance of an efficient monitoring programme in operational AW-IPM programmes incorporating the SIT, and the need for accurate marking of the sterile males, the development of transgenic strains that have both body markers and with sperm markers is commendable and may facilitate assessments of sterile to wild male ratios and mating status of wild females [25]. But certain disadvantages such as reduced competitiveness due to the presence of the transgene itself, or its particular insertion into the genome [41] must be addressed. Taking into account these limitations, efforts have been made to construct a transgenic VIENNA 8–1260 by using site specific integrase system [25,42] with additional transgenes at the same genomic position [43] considering that can potentially minimise the possibility of reduced fitness cost occurring due to particular insertion of the transgenes. Furthermore, Scolari et al. [25] assessed the positional effect of the transgenic insertion and its expression of fluorescent protein and showed no harmful effect on the sperm’s function. Our results do not support these observations and VIENNA 8–1260 showed lower productivity under semi mass-rearing conditions.

However, the development of transgenic strains with sperm markers has clearly potential and further research should be encouraged to develop new transgenic lines that have less negative effects on the competitiveness of the produced sterile males.
